# Drinking in transition: trends in alcohol consumption in Russia 1994-2004

**DOI:** 10.1186/1471-2458-10-691

**Published:** 2010-11-11

**Authors:** Francesca JA Perlman

**Affiliations:** 1 London School of Hygiene and Tropical Medicine, Keppel Street, London WC2E 7HT, UK

## Abstract

**Background:**

Heavy alcohol consumption is widespread in Russia, but studying changes in drinking during the transition from Communism has been hampered previously by the lack of frequent data. This paper uses 1-2 yearly panel data, comparing consumption trends with the rapid concurrent changes in economic variables (notably around the "Rouble crisis", shortly preceding the 1998 survey round), and mortality.

**Methods:**

Data were from 9 rounds (1994-2004) of the 38-centre Russia Longitudinal Monitoring Survey. Respondents aged over 18 were included (>7,000 per round). Trends were measured in alcohol frequency, quantity per occasion (by beverage type) and 2 measures of potentially hazardous consumption: (i) frequent, heavy spirit drinking (≥80 g per occasion of vodka or samogon and >weekly) (ii) consuming samogon (cheap home-distilled spirit). Trends in consumption, mean household income and national mortality rates (in the same and subsequent 2 years) were compared. Finally, in a subsample of individual male respondents present in both the 1996 and 1998 rounds (before and after the financial crash), determinants of changes in harmful consumption were studied using logistic regression.

**Results:**

Frequent, heavy spirit drinking (>80 g each time, ≥weekly) was widespread amongst men (12-17%) throughout, especially in the middle aged and less educated; with the exception of a significant, temporary drop to 10% in 1998. From 1996-2000, samogon drinking more than doubled, from 6% to 16% of males; despite a decline, levels were significantly higher in 2004 than 1996 in both sexes. Amongst women, frequent heavy spirit drinking rose non-significantly to more than 1% during the study. Heavy frequent male drinking and mortality in the same year were correlated in lower educated males, but not in women. Individual logistic regression in a male subsample showed that between 1996 and1998, those who lost their employment were more likely to cease frequent, heavy drinking; however, men who commenced drinking samogon in 1998 were more likely to be rural residents, materially poor, very heavy drinkers or pessimistic about their finances. These changes were unexplained by losses to follow-up.

**Conclusions:**

Sudden economic decline in late 1990s Russia was associated with a sharp, temporary fall in heavy drinking, and a gradual and persistent increase in home distilled spirit consumption, with the latter more common amongst disadvantaged groups. The correlation between heavy drinking and national mortality in lower educated men is interesting, but the timing of RLMS surveys late in the calendar year, and the absence of any correlation between drinking and the subsequent year's mortality, makes these data hard to interpret. Potential study limitations include difficulty in measuring multiple beverages consumed per occasion, and not specifically recording "surrogate" (non-beverage) alcohols.

## Background

Studying trends in alcohol consumption during the transition from Communism in Russia is potentially important in both adding to our understanding of the effects of rapid socioeconomic change on alcohol consumption, and seeing whether changes in harmful drinking were linked to the rapidly fluctuating death rates of that time.

A consistent association between economic change and trends in alcohol consumption in transitional Russia has not so far been shown. Whilst the sharp decline in vodka sales during the 2008 "credit crunch" reached the popular press [1], previous research has linked economic hardship variously to heavy drinking [2,3], abstention [3] or neither [4].

It is well known that heavy alcohol consumption, especially amongst men [4-7], is an important cause of premature death in Russia [8-10]. Indirect evidence also suggests that hazardous drinking played an important role in the increase and the fluctuations in mortality that followed the end of Communism in 1991, with a parallel between changes in all-cause mortality, deaths from alcohol poisoning [11,12] and causes of mortality partly attributable to alcohol [13], such as injuries and cardiovascular disease [11,14,15].

If alcohol were linked to the rapid changes in mortality, it would be reasonable to expect to see rapid changes in consumption, but it has not so far been shown that trends in alcohol consumption changed with death rates after 1991. Although population data from the mid-1980s at the time of Gorbachev's brief anti-alcohol campaign, showed declining consumption and rising life expectancy [16], the two were not linked subsequently. Limited data indicate that whilst population intake rose between 1989 and 1994, broadly reversing the decline of Gorbachev's campaign, mortality increased much further [13]. However, these consumption analyses were partly based on alcohol-related mortality, clearly limiting their value, and importantly could only approximate the widespread use of illegal alcohol after 1991 [13].

Two repeat surveys of individual level drinking showed a steady rise in consumption; however these studies were not sufficiently frequent to identify whether there were any rapid changes comparable to the fluctuations in GDP and mortality. In Novosibirsk, heavy consumption amongst males rose substantially between 1984/5 and 1988/9 (80 g or 120 g per occasion, and drinking 120 g more than weekly), but changed little between 1988/9 and 1994/5 [7,17]. From the 1990s, mean consumption in Karelia increased steadily from 45 g to 75 g per week (1992;1997; 2002; 2003), again without any marked fluctuations [18].

Importantly, however, these studies did not account for the wide range of beverages consumed in Russia, especially two classes of non-purchased alcohols: first "samogon" (home-distilled spirit) [19,20], and second the potentially harmful "surrogate" alcohols (e.g. colognes, medicines and industrial alcohols), which contain up to 95% alcohol [10,19]. More frequent measures of drinking could potentially identify more rapid alterations in alcohol consumption, and its relationship with changing economic conditions and mortality.

This paper has the advantage of using data collected every 1-2 years, from the Russia Longitudinal Monitoring Survey [21]. The data are used to examine changes in alcohol consumption during the Russian transition, and specifically to test the following hypotheses:

(i) During the years of the worst economic conditions, more people would consume cheaper beverages (including non-commercial spirits)

(ii) Fluctuations in potentially hazardous consumption (frequent, heavy spirit consumption and drinking non-commercial spirits) would be temporally associated with changes in national mortality rates.

## Methods

### Study design and subjects

The data were from Phase 2 of the Russia Longitudinal Monitoring Survey (RLMS), a panel study of households, and individuals within them, collected over 9 rounds between 1994 and 2004 (no survey took place in 1997 or 1999). This dataset has been described extensively elsewhere [21] and is considered the main information source on post-Soviet households. In brief, participants came from 38 population centres across the Russian Federation. St Petersburg and Moscow were included as the two largest metropolitan centres, and the remaining 36 districts, or primary sampling units (PSUs), were selected using probability proportional to size (PPS) after stratifying districts by socioeconomic criteria. Within the selected PSUs, urban and rural secondary sampling units (SSUs), census enumeration districts and villages respectively, were selected. From each SSU, 10 households were selected from the investigators' housing lists. The first dwelling was chosen at random, and the remainder at regular intervals. In subsequent rounds, households were re-interviewed, and those lost to follow up were replaced.

The overall response rate in the first round of Phase 2 (1994) was 84%, although in Moscow and St Petersburg it was approximately 67%. The investigators stated that the distribution of household size compared well to 1989 census data, as did sex, age and urban-rural distribution[21]. As households left the study, replacements were recruited. The turnover of individual respondents between each round was approximately 10-15%, although after the first (1994) round it was over 20% [22]. Individuals who left the study were more likely to be young, less educated, urban residents on higher incomes [8]. Further study details are given at: http://www.cpc.unc.edu/projects/rlms. The data were obtained from the study organisers at a time when they were freely available. Now, however, permission is required, and has been obtained.

### Measurements

#### Alcohol

Respondents were asked whether they had consumed alcohol within the last 30 days. For those who responded positively, there followed a single question on frequency. After this, respondents were asked which beverage they had consumed, and there was a question about typical quantity consumed per occasion for each beverage. This is effectively a modified version of the quantity-frequency approach, [23] and is somewhat limited by the questions, since quantity cannot be captured when more than one beverage type is consumed per occasion.

#### (a) Frequency

Respondents were asked whether they drank alcohol, and how frequently during the previous month. Frequency was initially divided into daily, 4-6 times a week, 2-3 times a week, once a week, 2-3 times a month, once in the last month and no alcohol in the last month. For some analyses it was collapsed into: none in the last month; between once in the last month and once a week; and more than once a week.

#### (b) Type of alcohol

Respondents were asked whether they had consumed beer, wine, fortified wine, vodka, home-distilled spirits ("samogon" in the Russian language questionnaire) in the last 30 days. They were also asked whether they had consumed any "other drinks", but this category was omitted from the analyses, since few people gave a positive answer (somewhat more women than men), and it was not possible to estimate the alcohol content, and was considered not to be useful, and unlikely to include "surrogate"(non-beverage) alcohols [10].

#### (c) Quantity of alcohol

For each beverage (beer, wine, fortified wine, vodka or home-distilled spirits), respondents reported the quantity usually consumed per occasion in grams (the measure normally used in Russia), which were converted into grams of pure alcohol using conversion factors based in part on concentrations stated on labels, but also on previous analyses of Russian drinks [19], as follows: beer 0.054;[24] dry or sparkling wine 0.142;[21] fortified wine 0.18;[21] vodka 0.44;[19] samogon 0.39[19].

Quantity was divided into 4 categories: very heavy episodic spirit consumption (160 g or more pure alcohol equivalent of samogon or vodka per occasion), heavy episodic spirit drinking (from 80 g up to 160 g of samogon or vodka per occasion), moderate consumption (less than 80 g spirits per occasion, or non-spirits only); and no alcohol. (Since quantity per occasion was measured separately for each beverage, if more than one beverage was consumed at a sitting, this could not be captured).

#### (d) Measures of potential "problem drinking" - heavy, frequent consumption; consuming samogon

Two measures were developed to reflect potentially harmful consumption patterns.

1) Frequent, heavy drinking, defined here as consuming at least 80 g spirits (vodka and/or samogon) per occasion, and drinking alcohol more than once a week. Frequent, heavy drinking is associated with increased mortality, particularly in Russia, [9,25,26].

2) Drinking samogon (any quantity). Samogon is a cheap non-purchased spirit, and whilst its health effects have not been studied, its drivers could be similar to the factors that drive the similarly inexpensive and potentially harmful surrogate alcohols[10].

### Other measures

3 age bands were used: 18-39; 40-59; 60 and over. Education was divided into incomplete secondary or less; complete secondary (general and/or technical); and higher. Area of residence was divided into: metropolitan (Moscow or St Petersburg), urban (other); and rural, and was defined by the investigators during the sampling process. Marital status was divided into married/cohabiting, single, divorced or widowed. Household material goods (colour television, VCR, car, washing machine, dacha) were combined into an asset score (0-5). Financial optimism was measured by asking "Do you think that in the next 12 months you and your family will live better than today, or worse?" (5-point scale).

For later ecological comparisons, the following measures were used:

Household income per person was calculated by dividing total household income (adjusted to the level of the 1994 rouble) by the square root of the number of occupants [27]. The age standardised mortality rate (ASMR), taken separately for men and women aged under 64 was based on routinely produced data from the Russian government [28]. Per capita alcohol consumption was taken from the World Drink Handbook, based on figures provided by the Russian government, predominantly trade data [29].

### Statistical analyses

#### (a) *Trends in consumption*

Consumption trends were studied separately by gender in respondents aged 18 and over (approximately 3-4,000 of each gender per round: Table 1). The prevalence of the following measures were age standardised to the earliest round (1994), and presented with 95% confidence intervals: drinking frequency; quantity per occasion of the beverage consumed in the greatest amount; heavy and frequent spirit consumption (more than once a week, and at least 80 g vodka or samogon per occasion); and beverage type. Trends in potentially harmful consumption were then compared by age group, education, and area of residence. All analyses were weighted (using household weights developed by the survey designers), and for clustering by site and for a variable that combined census district and family (in rural areas there was only one census district per site). Only clustering by site was possible in the subanalyses by education, district and age band, since numbers in the different strata were often small.

**Table 1:** Age, sex distribution of study population, and alcohol quantity and frequency by year-proportion (95% CI) standardised to 1994 study population

	1994	1995	1996	1998	2000	2001	2002	2003	2004
**Males**									

**Respondents-age (%)**									
18-29	898 (23.0)	781 (23.0)	774 (23.2)	844 (24.5)	934 (26.4)	1,045 (26.8)	1,112 (27.3)	1,158 (28.0)	1,161 (28.0)
30-39	935 (24.0)	772 (22.7)	751 (22.5)	754 (21.9)	704 (19.9)	777 (19.9)	753 (18.5)	808 (19.5)	830 (20.0)
40-49	805 (20.6)	702 (20.7)	703 (21.1)	708 (20.6)	747 (21.1)	818 (21.0)	886 (21.8)	874 (21.1)	858 (20.7)
50-59	572 (14.7)	509 (15.0)	482 (14.4)	456 (13.3)	427 (12.1)	465 (11.9)	516 (12.7)	539 (13.0)	586 (14.2)
60 and over	691 (17.7)	634 (18.7)	627 (18.8)	679 (19.7)	722 (20.4)	797 (20.4)	807 (19.8)	758 (18.3)	707 (17.1)
Total	3,901	3,398	3,337	3,441	3,534	3,902	4,074	4,137	4,142

**Frequency of drinking alcohol (% and 95% confidence interval)**									
Every day	2.6 (2.3-2.8)	3.4 (3.1-3.6)	3.0 (2.8-3.3)	2.4 (2.1-2.6)	3.4 (3.1-3.7)	3.3 (3.0-3.6)	4.4 (4.0-4.7)	3.4 (3.1-3.7)	3.1 (2.8-3.4)
4-6 times/week	2.8 (2.6-3.1)	3.1 (2.9-3.4)	3.1 (2.8-3.3)	2.3 (2.0-2.5)	3.4 (3.1-3.7)	3.9 (3.6-4.3)	4.2 (3.9-4.6)	3.5 (3.2-3.8)	3.3 (3.0-3.6)
2-3 times/week	13.2 (12.7-13.6)	13.7 (13.2-14.2)	13.6 (13.1-14.1)	10.1 (9.6-10.5)	13.3 (12.8-13.8)	15.1 (14.5-15.7)	15.5 (15.0-16.1)	15.4 (14.8-16.0)	15.0 (14.5-15.6)
once/week	19.3 (18.7-19.8)	18.6 (18.0-19.1)	19.2 (18.6-19.8)	16.5 (15.9-17.0)	18.1 (17.5-18.7)	17.4 (16.8-18.0)	17.9 (17.3-18.5)	17.8 (17.2-18.4)	17.7 (17.1-18.3)
2-3 times/month	22.7 (22.1-23.3)	22.8 (22.2-23.4)	21.4 (20.8-22.0)	24.5 (23.8-25.1)	20.7 (20.1-21.3)	21.0 (20.4-21.6)	17.4 (16.8-18.0)	18.8 (18.1-19.4)	17.3 (16.7-17.9)
once in last month	13.5 (13.0-13.9)	11.2 (10.7-11.6)	10.4 (10.0-10.8)	13.2 (12.7-13.6)	10.3 (9.9-10.8)	9.2 (8.7-9.6)	7.3 (6.9-7.7)	7.7 (7.3-8.1)	7.1 (6.7-7.6)
None	26.0 (25.4-26.6)	27.3 (26.6-27.9)	29.3 (28.7-29.9)	31.2 (30.5-31.9)	30.7 (30.0-31.4)	30.2 (29.4-30.9)	33.2 (32.5-34.0)	33.5 (32.7-34.3)	36.4 (35.6-37.2)

**Heavy drinking spirits - any frequency (% and 95% confidence interval)**									
> = 160 g	22.7 (22.1-23.2)	22.9 (22.4-23.5)	20.6 (20.0-21.1)	19.2 (18.6-19.8)	18.5 (17.9-19.1)	17.4 (16.8-18.0)	16.9 (16.3-17.5)	14.6 (14.0-15.1)	12.6 (12.1-13.1)
80-<160 g	28.4 (27.8-29.0)	27.2 (26.5-27.8)	27.2 (26.6-27.9)	26.9 (26.3-27.5)	25.2 (24.5-25.8)	26.7 (26.0-27.4)	23.9 (23.2-24.6)	25.2 (24.5-25.9)	23.9 (23.3-24.6)

**Frequent, heavy drinking spirits - more than weekly (% and 95% confidence interval)**									
> = 160 g occasion & > wkly	7.2 (6.8-7.6)	7.4 (7.0-7.8)	7.3 (7.0-7.7)	5.0 (4.7-5.3)	6.8 (6.4-7.1)	8.0 (7.6-8.4)	8.2 (7.7-8.6)	6.6 (6.2-7.0)	6.0 (5.6-6.3)
80-<160 g occasion & > wkly	6.8 (6.4-7.1)	6.8 (6.4-7.2)	7.3 (6.9-7.7)	5.1 (4.8-5.4)	6.1 (5.7-6.5)	7.8 (7.4-8.3)	7.6 (7.2-8.0)	7.7 (7.2-8.1)	7.6 (7.1-8.0)

**Females**									

**Respondents-age (%)**									
18-29	1,006 (20.3)	903 (20.0)	946 (21.1)	983 (21.5)	1,097 (22.9)	1,279 (23.6)	1,299 (23.3)	1,346 (23.7)	1,319 (23.2)
30-39	1,030 (20.8)	869 (19.3)	812 (18.1)	781 (17.1)	755 (15.7)	846 (15.6)	881 (15.8)	917 (16.2)	961 (16.9)
40-49	853 (17.2)	829 (18.4)	819 (18.3)	877 (19.2)	938 (19.5)	1,042 (19.2)	1,055 (18.9)	1,041 (18.3)	1,024 (18.0)
50-59	740 (14.9)	633 (14.1)	623 (13.9)	580 (12.7)	592 (12.3)	664 (12.3)	724 (13.0)	806 (14.2)	856 (15.1)
60 and over	1,333 (26.9)	1,272 (28.2)	1,276 (28.5)	1,358 (29.7)	1,419 (29.6)	1,585 (29.3)	1,612 (28.9)	1,569 (27.6)	1,518 (26.7)
Total	4,962	4,506	4,476	4,579	4,801	5,416	5,571	5,679	5,678

**Frequency of drinking alcohol (% and 95% confidence interval)**									
Every day	0.5 (0.5-0.5)	0.6 (0.5-0.6)	0.5 (0.4-0.5)	0.3 (0.2-0.3)	0.6 (0.6-0.7)	0.6 (0.5-0.6)	0.6 (0.6-0.7)	0.7 (0.6-0.7)	0.7 (0.6-0.7)
4-6 times/week	0.3 (0.3-0.4)	0.4 (0.3-0.4)	0.4 (0.3-0.4)	0.3 (0.3-0.3)	0.4 (0.3-0.4)	0.5 (0.5-0.6)	0.5 (0.4-0.5)	0.7 (0.6-0.7)	0.7 (0.6-0.7)
2-3 times/week	1.9 (1.8-2.0)	2.2 (2.1-2.3)	2.3 (2.2-2.4)	1.9 (1.8-2.0)	2.6 (2.5-2.7)	3.5 (3.3-3.6)	3.2 (3.1-3.3)	3.3 (3.2-3.4)	3.4 (3.3-3.5)
once/week	5.9 (5.8-6.0)	7.1 (6.9-7.2)	6.0 (5.9-6.2)	6.0 (5.8-6.1)	6.4 (6.2-6.5)	7.9 (7.8-8.1)	9.2 (9.0-9.4)	8.5 (8.3-8.7)	7.8 (7.6-8.0)
2-3 times/month	16.4 (16.2-16.6)	16.3 (16.1-16.5)	15.4 (15.1-15.6)	15.8 (15.6-16.0)	17.3 (17.1-17.6)	16.9 (16.6-17.1)	16.6 (16.3-16.8)	17.4 (17.2-17.7)	17.4 (17.1-17.6)
once in last month	19.8 (19.6-20.0)	17.8 (17.6-18.0)	18.9 (18.7-19.1)	18.7 (18.5-19.0)	16.7 (16.4-16.9)	16.2 (15.9-16.4)	14.9 (14.6-15.1)	14.5 (14.3-14.8)	14.3 (14.1-14.5)
None	55.2 (54.9-55.4)	55.6 (55.3-55.9)	56.6 (56.3-56.9)	57.0 (56.7-57.3)	56.1 (55.8-56.4)	54.5 (54.2-54.8)	55.0 (54.7-55.4)	54.9 (54.6-55.2)	55.8 (55.4-56.1)

**Heavy drinking spirits - any frequency (% and 95% confidence interval)**									
> = 160 g	1.5 (1.4-1.6)	2.3 (2.2-2.4)	1.8 (1.7-1.9)	1.7 (1.6-1.7)	1.4 (1.3-1.5)	1.7 (1.6-1.7)	1.3 (1.3-1.4)	1.1 (1.0-1.1)	1.1 (1.0-1.2)
80-<160 g	7.2 (7.1-7.4)	7.7 (7.5-7.8)	7.0 (6.9-7.2)	7.4 (7.2-7.5)	6.1 (6.0-6.3)	7.1 (6.9-7.3)	7.2 (7.0-7.4)	6.8 (6.7-7.0)	6.8 (6.7-7.0)

**Frequent, heavy drinking spirits - more than weekly (% and 95% confidence interval)**									
> = 160 g occasion & > wkly	0.2 (0.2-0.3)	0.6 (0.5-0.6)	0.4 (0.4-0.5)	0.3 (0.3-0.4)	0.4 (0.4-0.4)	0.6 (0.6-0.7)	0.5 (0.5-0.6)	0.3 (0.3-0.4)	0.3 (0.3-0.4)
80-<160 g occasion & > wkly	0.7 (0.7-0.8)	0.7 (0.7-0.8)	0.7 (0.7-0.8)	0.5 (0.5-0.6)	0.6 (0.5-0.6)	0.9 (0.8-0.9)	1.3 (1.2-1.3)	1.0 (0.9-1.1)	1.0 (0.9-1.1)

To test the hypotheses set out in the introduction, trends in potentially hazardous alcohol consumption were compared with mean household income and age standardised mortality rates, as well as with ecological per capita alcohol consumption. Respondents aged under 60 were included, since they experienced the greatest fluctuations in mortality, and were stratified into 2 educational groups (incomplete secondary vs complete secondary or higher. The associations were first compared graphically, and then by examining correlations (a) over the whole study (b) during 1996-2000 (when changes in consumption were significant). Correlation analyses also included lagged mortality (deaths over the subsequent 2 years), to test for a delayed effect of drinking on mortality.

#### (b) *Individual level multivariate analyses of changes in drinking between 1996-8*

Preliminary analyses had shown that in men, frequent, heavy drinking declined, and samogon consumption rose significantly between 1996 and 1998 (before and after the 1998 financial crash). To study the determinants of these changes amongst individual participants, multivariate logistic regression analyses were performed on a subsample of male participants present in both 1996 and 1998:

(i) Commencing samogon consumption in 1998 [baseline: drinking alcohol (but not samogon) in 1996; outcome: drinking samogon in 1998.]

(ii) Ceasing heavy frequent spirit consumption in 1998 [baseline: heavy, frequent spirit drinking (>80 g samogon or vodka, ≥ weekly) in 1996; outcome: negative for this measure in 1998].

3 models were used, adjusted as follows: Model 1 - age; Model 2 - age, urban/rural, marital status; Model 3 - age, urban/rural, marital status, education, asset score.

## Results

### (a) *Trends in consumption*

During the study period, frequent drinking rose in men, with more than weekly drinking rising from 17% to 21%. However, there was a significant decline in heavy male drinking: consuming ≥160 g per occasion declined from 22% to 12%. The proportion of frequent, heavy male drinkers did not change significantly between 1994 and 2004, with a steady 13-14% drinking more than weekly and consuming ≥80 g per occasion. In women, drinking more than weekly rose from 2% to 4%. The proportion of female heavy drinkers changed very little, with approximately 8% of women drinking ≥80 g per occasion throughout the study. Frequent, heavy drinking rose non-significantly amongst women to over 1%. Superimposed on these changes, in 1998 there was a transient decline in frequent drinking in both sexes, and in frequent, heavy drinking amongst men (Table 1).

Frequent, heavy drinking in most rounds was significantly more common amongst men aged 40-59 years than in older and younger men, but there was a steady increase amongst the youngest age group. Initially, this pattern was more common in Moscow and St Petersburg, but a decline in these metropolitan areas meant that regional variations had almost disappeared by 2004 (Table 2). The period that included the major financial "Rouble" crisis of August1998 was followed by a sharp decline in frequent, heavy male drinking (Table 1). This decline was significant in men in all 3 age groups, non-metropolitan residents and in all educational groups (Table 2) although levels had recovered almost completely by 2000. Amongst women, this decline was significant in those with less than tertiary education, urban women, and those aged 40-59.

**Table 2:** Trends in prevalence (%) of heavy and frequent drinking and samogon consumption by age group, area and education

		1994	1995	1996	1998	2000	2001	2002	2003	2004
**Males**										
Frequent, heavy spirit cons.*										
**Education**	Incompl 2ry	17.9 (16.9-18.8)	16.5 (15.4-17.5)	16.1 (15.3-17.0)	**11.0 (10.2-11.8)**	**14.6 (13.5-15.7)**	18.0 (16.8-19.2)	20.8 (19.7-21.8)	15.3 (14.3-16.3)	17.4 (16.3-18.6)
	Compl. 2ry	14.1 (13.1-15.0)	14.6 (13.5-15.6)	14.7 (13.5-15.9)	**10.9 (9.8-11.9)**	**14.3 (13.1-15.5)**	16.3 (15.3-17.4)	15.4 (14.3-16.5)	14.5 (13.4-15.5)	13.6 (12.6-14.5)
	Higher	12.0 (11-13.1)	10.9 (9.8-12)	13.1 (11.9-14.3)	**9.2 (7.9-10.4)**	**11.0 (9.9-12.1)**	13.6 (12.3-15.0)	13.6 (12.2-15.0)	13.8 (12.4-15.3)	10.9 (9.9-11.9)
**Area**	Urban	13.3 (12.6-13.9)	13.3 (12.7-14.0)	14.6 (14.0-15.3)	**9.9 (9.4-10.5)**	**13.0 (12.3-13.7)**	17.0 (16.2-17.7)	17.5 (16.7-18.3)	14.6 (13.8-15.3)	13.5 (12.8-14.2)
	Rural	13.8 (12.8-14.8)	14.5 (13.5-15.5)	13.2 (12.3-14.1)	**8.7 (7.9-9.5)**	**10.4 (9.6-11.3)**	11.4 (10.5-12.3)	12.2 (11.4-13.0)	12.9 (11.9-13.9)	14.3 (13.2-15.4)
	Mosc/St Pete	16.8 (14.6-18.9)	20.1 (17.6-22.7)	20.1 (17.4-22.8)	**16.7 (14.0-19.5)**	**23.4 (19.6-27.2)**	17.8 (15.4-20.3)	13.3 (11.2-15.3)	13.9 (11.5-16.3)	11.2 (9.2-13.3)
**Age band**	18-39	12.7 (11.9-13.5)	14.4 (13.5-15.3)	13.7 (12.8-14.6)	**9.6 (8.8-10.5)**	**12.5 (11.5-13.5)**	17.8 (16.6-19.0)	17.4 (16.0-18.8)	14.0 (12.8-15.2)	14.5 (13.6-15.5)
	40-59	18.5 (17.5-19.4)	16.2 (15.2-17.1)	18.5 (17.4-19.6)	**13.3 (12.4-14.1)**	**14.0 (12.9-15.2)**	16.7 (15.5-17.9)	16.2 (15.2-17.2)	18.2 (17.0-19.4)	15.8 (14.8-16.9)
	60 and over	11.4 (10.3-12.5)	12.2 (11.0-13.4)	11.3 (9.8-12.8)	**6.5 (5.4-7.5)**	**12.4 (11.2-13.7)**	11.6 (10.5-12.7)	13.6 (12.3-15.0)	11.8 (10.3-13.2)	11.7 (10.0-13.5)

**Samogon**										
**Education**	Incompl 2ry	7.8 (7.0-8.5)	8.4 (7.6-9.2)	8.4 (7.5-9.2)	**15.5 (14.3-16.7)**	**23.9 (22.5-25.3)**	22.2 (20.8-23.6)	20.5 (18.8-22.2)	18 (16.6-19.5)	19 (17.3-20.7)
	Compl. 2ry	6.7 (5.8-7.6)	4.6 (3.9-5.3)	6.5 (5.5-7.4)	**12.1 (11.0-13.3)**	**15.3 (13.7-16.8)**	16.7 (15.1-18.4)	15.9 (14.5-17.4)	13.2 (11.7-14.7)	11.8 (10.6-13.0)
	Higher	2.9 (2.4-3.5)	2.6 (1.8-3.4)	3.4 (2.7-4.1)	**7.7 (6.4-9.1)**	**10.3 (8.7-12)**	9.3 (7.6-10.9)	9.4 (7.5-11.4)	8.7 (7.0-10.4)	6.0 (4.6-7.5)
**Area**	Urban	4.5 (4.1-4.8)	3.8 (3.5-4.2)	5.0 (4.6-5.3)	**9.8 (9.2-10.3)**	**13.6 (12.9-14.3)**	14.1 (13.5-14.8)	14.0 (13.3-14.6)	11.4 (10.8-12)	10.3 (9.7-10.9)
	Rural	10.9 (10.0-11.7)	10.5 (9.6-11.3)	10.7 (9.9-11.6)	**18.9 (17.8-20.0)**	**26.5 (25.2-27.7)**	27.9 (26.6-29.2)	24.4 (23.2-25.6)	22.5 (21.4-23.7)	18.3 (17.3-19.4)
	Mosc/St Pete	1.4 (0.8-2.1)	0.5 (0.0-0.9)	0.8 (0.3-1.3)	**2.6 (1.4-3.7)**	**2.9 (1.7-4.2)**	2.3 (1.4-3.3)	2.4 (1.5-3.4)	2.3 (1.0-3.5)	2.8 (1.6-4.0)
**Age band**	18-39	4.9 (4.3-5.4)	4.6 (4-5.3)	5.0 (4.4-5.7)	**10.9 (10.0-11.8)**	**16.4 (14.9-17.9)**	14.8 (13.4-16.1)	13.6 (12.0-15.1)	11.8 (10.3-13.2)	9.9 (8.5-11.2)
	40-59	7.1 (6.3-8.0)	6.1 (5.3-6.9)	7.2 (6.4-8.1)	**11.1 (9.8-12.4)**	**16.8 (15.2-18.3)**	16 (14.5-17.5)	15.3 (13.7-16.8)	15 (13.5-16.5)	12.7 (11.3-14)
	60 and over	8.0 (6.8-9.1)	6.2 (5.0-7.4)	7.7 (6.4-9.0)	**12.3 (10.8-13.8)**	**18.2 (16.2-20.1)**	20.7 (18.6-22.8)	19.1 (17.1-21.1)	16.7 (14.6-18.7)	16.1 (14.0-18.2)

**Female**										
Frequent, heavy spirit cons.*										
**Education**	Incompl 2ry	1.8 (1.6-1.9)	2.3 (2.1-2.5)	2.1 (1.9-2.3)	**1.2 (1.0-1.3)**	**1.6 (1.4-1.7)**	2.7 (2.5-2.9)	3.5 (3.2-3.9)	1.9 (1.7-2.1)	2.9 (2.7-3.2)
	Compl. 2ry	1.3 (1.0-1.6)	1.1 (0.8-1.4)	1.5 (1.1-1.9)	**0.7 (0.5-1.0)**	**1.2 (1.0-1.5)**	1.6 (1.2-1.9)	1.5 (1.2-1.7)	1.2 (0.9-1.4)	1.3 (1.0-1.6)
	Higher	0.7 (0.5-0.9)	0.8 (0.6-1.0)	0.8 (0.6-1.0)	**0.8 (0.6-1.0)**	**0.7 (0.5-0.9)**	1.1 (0.9-1.3)	1.5 (1.3-1.7)	1.2 (1.0-1.4)	0.9 (0.8-1.1)
**Area**	Urban	0.9 (0.8-1.0)	1.2 (1.1-1.3)	1.1 (1.0-1.2)	**0.7 (0.6-0.8)**	**0.9 (0.8-1.0)**	1.3 (1.2-1.4)	1.8 (1.7-2.0)	1.3 (1.1-1.4)	1.3 (1.2-1.5)
	Rural	0.6 (0.5-0.7)	0.8 (0.7-0.9)	0.7 (0.6-0.8)	**0.9 (0.8-1.1)**	**0.9 (0.8-1.1)**	1.6 (1.4-1.8)	1.4 (1.2-1.5)	1.5 (1.3-1.6)	1.5 (1.3-1.6)
	Mosc/St Pete	2.1 (1.7-2.5)	3.0 (2.4-3.7)	1.8 (1.3-2.3)	**1.9 (1.3-2.4)**	**0.4 (0.2-0.7)**	1.7 (1.2-2.2)	1.9 (1.4-2.4)	1.3 (0.8-1.8)	1.2 (0.8-1.5)
**Age band**	18-39	1.4 (1.1-1.6)	1.5 (1.2-1.8)	1.2 (1.0-1.5)	**1.1 (0.8-1.4)**	**1.5 (1.2-1.8)**	2.5 (2.0-2.9)	3.3 (2.8-3.8)	2.0 (1.7-2.4)	1.8 (1.4-2.2)
	40-59	0.8 (0.7-1.0)	1.7 (1.4-2.0)	1.3 (1.1-1.4)	**0.7 (0.5-0.8)**	**1.2 (1.0-1.5)**	1.6 (1.4-1.8)	1.4 (1.1-1.6)	1.6 (1.4-1.9)	1.3 (1.1-1.6)
	60 and over	0.2 (0.1-0.2)	0.6 (0.5-0.7)	0.9 (0.8-1.0)	**0.7 (0.6-0.7)**	**0.2 (0.2-0.3)**	0.5 (0.5-0.6)	0.5 (0.4-0.5)	0.3 (0.2-0.3)	0.8 (0.7-0.9)

**Samogon**										
**Education**	Incompl 2ry	1.7 (1.6-1.9)	1.4 (1.3-1.6)	2.1 (1.9-2.3)	**4.1 (3.8-4.3)**	**7.8 (7.4-8.2)**	8.1 (7.7-8.5)	7.2 (6.7-7.6)	5.9 (5.5-6.3)	5.0 (4.7-5.3)
	Compl. 2ry	1.3 (0.9-1.8)	0.7 (0.5-0.8)	1.2 (0.9-1.4)	**3.7 (3.2-4.2)**	**4.7 (4.0-5.3)**	4.9 (4.1-5.7)	4.4 (3.6-5.1)	2.8 (2.2-3.4)	2.9 (2.3-3.5)
	Higher	0.8 (0.6-1.0)	0.5 (0.4-0.7)	0.5 (0.4-0.7)	**2.2 (1.8-2.6)**	**3.3 (2.8-3.7)**	3.3 (2.9-3.7)	3.1 (2.6-3.6)	2.2 (1.8-2.6)	1.5 (1.2-1.8)
**Area**	Urban	0.9 (0.8-0.9)	0.7 (0.7-0.8)	1.1 (1.0-1.2)	**2.5 (2.4-2.6)**	**4.2 (4.0-4.4)**	4.3 (4.1-4.5)	4.1 (3.9-4.3)	2.8 (2.6-3.0)	2.2 (2.0-2.3)
	Rural	1.5 (1.4-1.7)	1.6 (1.4-1.7)	1.8 (1.6-2.0)	**6.5 (6.2-6.9)**	**7.4 (7.0-7.7)**	8.2 (7.8-8.6)	7.5 (7.2-7.9)	5.4 (5.0-5.7)	5.6 (5.2-6.0)
	Mosc/St Pete	0.2 (0.1-0.3)	0	0	**1.4 (0.8-1.9)**	**0.7 (0.4-1.1)**	1.1 (0.8-1.5)	1.0 (0.7-1.4)	0.9 (0.5-1.3)	0
**Age band**	18-39	0.8 (0.6-1.0)	0.9 (0.7-1.2)	0.9 (0.7-1.1)	**3.6 (3.0-4.1)**	**5.1 (4.3-5.9)**	5.0 (4.3-5.8)	4.8 (3.9-5.7)	3.5 (2.9-4.1)	2.6 (2.0-3.1)
	40-59	1.4 (1.2-1.6)	0.9 (0.7-1.1)	1.4 (1.2-1.6)	**4.1 (3.6-4.5)**	**6.2 (5.6-6.8)**	6.0 (5.4-6.7)	4.9 (4.2-5.5)	2.9 (2.5-3.3)	3.6 (3.1-4.2)
	60 and over	0.9 (0.8-1.0)	0.8 (0.7-0.9)	1.0 (0.9-1.1)	**2.7 (2.5-2.9)**	**3.2 (3.0-3.4)**	3.6 (3.4-3.8)	4.3 (4.0-4.5)	3.3 (3.1-3.6)	2.6 (2.4-2.8)

**Figure 1:**
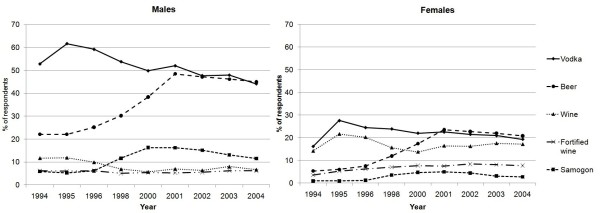
**Trends in percentage of Russian men and women consuming alcohol beverages, by beverage type, 1999-2004**.

There were important changes in the beverages consumed (Figure 1). Between 1995 and 2004, the percentage of vodka consumers declined from 61% to 44% in men and 27% to 19% amongst women. The proportion of wine drinkers also fell substantially during the earlier rounds. Conversely, between 1994 and 2001, the percentage of beer drinkers increased dramatically from 22% to nearly 50% of men and 5% to 20% of women, and samogon consumers increased from 5% to 16% of men and from less than 1% to nearly 5% of women. The greatest rises occurred between 1996-1998 (the year of the financial crisis) and between 1998-2000.

Samogon consumption in both sexes rose significantly in each age and educational group, and in non-metropolitan urban and rural residents (and metropolitan men), and despite a decline after 2002, samogon drinking remained significantly more common than in 1994. Educational and urban-rural gradients in the prevalence of samogon consumption widened throughout the study (Table 2).

**Figure 2:**
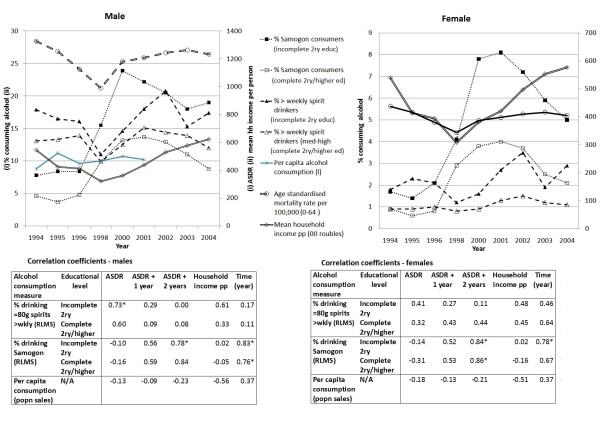
**Alcohol consumption (RLMS), age standardised death rates **[28], **per capita alcohol consumption**[56]**and mean household income 1994-2004**.

Figure 2 compares changes in drinking, mean household income, alcohol sales data and mortality. There were correlations between heavy, frequent consumption, household income and mortality in the same year in men, strongest amongst the least educated. However, these correlations were not present in women, and samogon consumption was not correlated with mean income or mortality over the whole study period. There was no lagged effect of correlation between alcohol and mortality in the following year. There seemed to be some inconsistencies in the relationships between consumption, mortality and mean income across the study period. Per capita consumption based on alcohol sales showed no similarities to the trends in harmful drinking measured in the survey, or with mortality.

### (b) *Individual level changes between 1996-8-multivariate analyses*

Between 1996 and 1998, 90 out of 593 male frequent, heavily drinkers stopped doing so. During the same period, 213 out of 3419 male non-samogon drinkers started drinking samogon. Determinants of these consumption changes at individual level (using logistic regression) are shown in Table 3.

**Table 3:** Determinants of changes in drinking 1996-8 amongst 1996 male respondents (a) Samogon in 1998 amongst 1996 drinkers (non-samogon consumers) (b) Non-heavy frequent spirit (vodka/samogon) drinking in 1998 amongst frequent heavy spirit drinkers (1996) (RLMS)

	Drinking samogon in 1998 *n = 213 *[baseline: 1996 drinkers (non-samogon consumers) *n = 3,419*] Odds ratio (95% CI)			Non frequent heavy spirit drinking in 1998 *n = 98 *[baseline: 1996 frequent heavy spirit drinkers (≥80 g & > wkly) *n = 593*] - Odds ratio (95% CI)		
	**Model 1** **(age adjusted)**	**Model 2 (age, urban/rural, marital)**	**Model 3 ( = Model 2 + education, asset)**	**Model 1** **(age adjusted)**	**Model 2 (age, urban/rural, marital)**	**Model 3 ( = Model 2 + education, asset)**

**Age**						
18-39	1	1	1	1	1	1
40-59	1.29 (0.91-1.83)	1.39 (0.95-2.02)	1.35 (0.93-1.97)	**0.37 (0.22-0.60)**	**0.36 (0.22-0.61)**	**0.32 (0.19-0.55)**
60 and over	**2.27 (1.49-3.44)**	**2.49 (1.58-3.93)**	**2.18 (1.38-3.45)**	0.49 (0.23-1.02)	0.51 (0.23-1.10)	0.43 (0.18-1.00)
**Education**						
Higher	1	1	1	1	1	1
Complete secondary	**1.88 (1.26-2.79)**	**1.62 (1.07-2.46)**	1.41 (0.92-2.14)	0.86 (0.50-1.48)	0.90 (0.52-1.56)	0.88 (0.50-1.54)
Incomplete secondary	1.36 (0.88-2.09)	1.05 (0.66-1.65)	0.87 (0.55-1.39)	1.28 (0.71-2.32)	1.65 (0.86-3.17)	1.60 (0.82-3.12)
**Area**						
Urban	1	1	1	1	1	1
Rural	**1.88 (1.35-2.61)**	**1.88 (1.35-2.62)**	**1.46 (1.03-2.08)**	0.72 (0.42-1.23)	0.68 (0.40-1.17)	0.55 (0.30-1.00)
Moscow St P	**0.25 (0.08-0.81)**	**0.26 (0.08-0.83)**	**0.26 (0.08-0.85)**	0.64 (0.31-1.29)	0.64 (0.32-1.31)	0.66 (0.32-1.36)
**Marital status**						
Married	1	1	1	1	1	1
Single	1.59 (0.95-2.67)	1.54 (0.91-2.61)	1.54 (0.91-2.61)	1.55 (0.53-4.56)	1.65 (0.56-4.90)	1.57 (0.53-4.67)
Divorced	**1.88 (1.09-3.24)**	1.64 (0.93-2.91)	1.64 (0.93-2.91)	0.92 (0.47-1.80)	1.39 (0.64-2.99)	1.32 (0.61-2.89)
Widowed	0.63 (0.22-1.84)	0.68 (0.23-2.01)	0.68 (0.23-2.01)	0.92 (0.24-3.54)	0.74 (0.19-2.91)	0.66 (0.16-2.65)
**Optimism over financial situation (1-5 high-low)**	**1.30 (1.11-1.52)**	**1.26 (1.07-1.49)**	**1.23 (1.05-1.46)**	0.97 (0.78-1.21)	0.98 (0.78-1.23)	0.97 (0.77-1.22)
**Asset score (0-5 low-high)**	**0.73 (0.65-0.82)**	**0.76 (0.66-0.87)**	**0.76 (0.66-0.87)**	1.00 (0.86-1.16)	0.96 (0.80-1.15)	0.96 (0.80-1.15)
**Decline in asset score 1996-98 (yes vs no)**	0.64 (0.31-1.30)	0.89 (0.59-1.36)	1.06 (0.69-1.64)	1.27 (0.70-2.31)	1.21 (0.66-2.21)	1.28 (0.68-2.41)
**Employment status 1996-98**						
Working 96 & 98	1	1	1	1	1	1
Working 96; not working 98	**0.27 (0.17-0.45)**	**0.26 (0.16-0.44)**	**0.24 (0.14-0.39)**	**5.13 (2.55-10.32)**	**5.56 (2.67-11.56)**	**5.52 (2.64-11.51)**
Not working 96;working 98	1.23 (0.64-2.35)	1.20 (0.61-2.34)	1.08 (0.55-2.12)	0.66 (0.25-1.74)	0.61 (0.23-1.63)	0.60 (0.22-1.64)
Not working 96 or 98	0.87 (0.58-1.30)	0.74 (0.48-1.14)	**0.61 (0.39-0.95)**	1.72 (0.99-2.99)	1.65 (0.92-2.94)	1.61 (0.89-2.94)
**Drinking pattern - amount per occasion (g) & frequency**						
<80 g ≤ weekly	1	1	1	-	-	-
80-160 g ≤ weekly	**1.65 (1.01-2.69)**	1.51 (0.92-2.51)	1.41 (0.85-2.33)	-	-	-
>160 g ≤ weekly	1.64 (0.97-2.79)	1.47 (0.85-2.53)	1.32 (0.76-2.28)	-	-	-
<80 g >weekly	1.05 (0.42-2.64)	0.92 (0.34-2.51)	0.90 (0.33-2.47)	-	-	-
80-160 g >weekly	0.82 (0.39-1.74)	0.89 (0.42-1.92)	0.85 (0.39-1.83)	-	-	-
>160 g >weekly	**2.60 (1.47-4.58)**	**2.26 (1.24-4.10)**	**2.04 (1.12-3.73)**	-	-	-

Men who stopped frequent, heavy drinking in 1998 were more likely to have been newly unemployed in that year, younger or living outside the main cities. In contrast, men who started to drink samogon in 1998 were significantly more likely to be older (over 60), pessimistic over family finances, secondary educated or less, very heavy drinkers (≥160 g and > wkly) or rural (vs urban) residents. However, metropolitan (vs urban) residents, men with fewer material assets or who became unemployed between 1996-8 were less likely to commence drinking samogon.

## Discussion

### Discussion of findings

The high levels of male alcohol consumption in RLMS were as expected, and broadly consistent with, and sometimes higher than in, other contemporaneous Russian studies [4,7,30], allowing for variations in the measures used. Just over half the men in RLMS drank at least 80 g spirits per sitting, comparable with 51% in Novosibirsk in 1994/5 [7], and higher than 44% in the 1996 New Russia Barometer[4] and 30% in Novosibirsk in 1998/9 [30]. Considerably more men drank 160 g spirits per occasion (20% in 1996 and 19% in 1998 in RLMS) than in the 1996 NRB (14%) [4]. A similar proportion of RLMS respondents had not drunk in the last month (26-34% of men and 55% of women) to Novosibirsk in 1994 (35% of men and 60% of women) [7] and 1999-2000 (20% and 70%) [30], and in the 1996 NRB (29% of men and 70% of women) [4]. The 15% of RLMS males drinking at least 80 g and more than weekly, was higher than 10% in Novosibirsk in 1989-90, although the questions differed [30]. It is possible that including samogon may account for the slightly higher rates in some instances in RLMS.

These findings illustrate the complexity of the trends in alcohol consumption during the Russian transition. The rise in frequent drinking coincides with findings in Karelia [31] and suggests that the upward trend of the late 1980s in Novosibirsk has continued [7]. In contrast, the quantity of alcohol consumed per occasion did not increase during either of these studies [7,31], and actually declined during RLMS. It is possible that this could be related to a rise in non-spirit consumption, such as beer.

Again, the pattern of beverage consumption showed some similarities to another study. In 2003, 48% of men in RLMS had consumed purchased spirits, 48% beer and 8% wine within the previous month. Despite variations in question wording, consumption amongst the male controls in the Izhevsk study, the only suitable comparator, was of a similar order: 24% drank spirits at least once a week, and 54% once a month or less; such figures were 48% and 30% for beer, and 7% and 33% for wine respectively [32]. Unfortunately, however, surrogate alcohols, consumed by 7% of men in Izhevsk, were not measured in RLMS.

Regarding the hypotheses at the start of this paper, there is some evidence to support the first, that more people would consume inexpensive spirits at a time of financial hardship. Two distinct changes in consumption in 1998 followed the "Rouble" economic crisis [33] two months prior to the survey of that year. The first, as hypothesised, was an increase in samogon consumption. The second, less expected change was a reduction in the proportion of frequent, heavy drinkers.

The marked temporary decline in frequent heavy drinking, similar to a fall in Taganrog between 1993/4 and 1998 [34], was closely associated with job loss in RLMS, and frequent heavy consumption resumed rapidly in 2000-2001 with improved economic wellbeing. It is possible that this first group consists of previously employed, non-dependent heavy drinkers who were able to stop and recommence drinking in response to their circumstances.

In contrast, a much wider range of factors were associated with starting to drink samogon in 1998 in men who were not doing so in 1996. The particularly high risk amongst the heaviest drinkers suggests that alcohol dependence (combined with affordability) was a likely precipitant, and the greater increase amongst rural residents could be related to easier access. For the materially poor, affordability was a factor, partly explaining the effect of low education. The independent effect of low optimism suggests that psychological influences may be important in drinking decisions, although more detailed research is clearly required. Those who started to drink appear to constitute a second and separate group, who were more multiply disadvantaged.

The differences in trends between the 2 drinking patterns persisted. Whilst frequent, heavy drinking resumed in 2001, consumption of relatively inexpensive samogon and beer rose further, declining later and more gradually. This pattern suggests that the drivers of samogon consumption are not simply financial, although low cost is undoubtedly important [20]. Compared with vodka, at approximately 60 roubles [19,35], samogon costs approximately 10-15 roubles per half-litre to buy, and 4-5 roubles to manufacture [36], and bartering is widespread [19,36]. Preference for home distilling and fear of poisoning by counterfeit vodkas may also contribute to preference for home-distilled liquor [20,37]. The high prevalence of consumption indicates that samogon is now established in mainstream society, and no longer confined to older rural dwellers [20]. The sustained rise in beer drinking could also be explained by low cost and perceived safety, but the relative ease of purchase and public consumption prior to restrictive legislation in 2006 may also be important factors [38].

The second initial hypothesis appeared to be partly supported by the correlation between national mortality rates and rates of frequent, heavy drinking amongst the least educated men in the same year [11,14,39], although such associations were not demonstrated in more educated men, female heavy drinkers, or for samogon consumption. However, caution is required in interpreting these data, for several reasons. First, and most importantly, RLMS surveys occur in the final quarter of each calendar year, after the majority of deaths that contribute to that year's ASDR have taken place, and it is surprising that potentially hazardous drinking was not correlated with the following year's ASDR. Second, it is hard to identify and account for the role of relevant confounders. Mean income changes were also correlated with mortality; and elsewhere employment variables have been shown to fluctuate during the transition [40]. Third, surrogate alcohol consumption was not measured [10], although it is plausible that trends were similar to those of samogon, a similarly inexpensive non-commercial spirit [19]. Fourth, there was no survey in either 1997 or 1999, although it seems most likely that the consumption changes between 1996-8 were related to the most significant event, the "rouble crisis" of autumn 1998.

Apart from the trends, the overall levels of frequent, heavy consumption, were more common in two groups that experienced higher excess mortality during the transition. The first was the least educated [8,14,41]. The educational gradient in frequent, heavy drinking in RLMS remains wide, and was consistent with that in Taganrog in 1993[42] and Izhevsk (beverage and non-beverage alcohols) in 2007 [10]. The second group at greater risk of excess mortality was middle-aged men. Heavy, frequent consumption was also more common in this group, both here and in 2 other studies [34,43], although the increase in younger men must also be of concern.

This study showed two other important findings. First, the decline in heavy male alcohol consumption in Moscow and St Petersburg contributed to regional convergence, and could indicate a move amongst metropolitan residents towards more Western drinking patterns [44].

Second, consumption trends differed by gender. Whilst female respondents drank less than men, it was notable that heavy, frequent drinking almost doubled in women, and samogon consumption increased more markedly. At the same time, however, a higher proportion of women drank wine, and this proportion rose. There appears to be a contrast between the majority of women who either do not drink, or who have more Western consumption patterns, and a small minority of hazardous women drinkers. Younger women drank more than their older counterparts, and interestingly, female smoking rates also rose most steeply in this age group [45], suggesting that more young women are adopting risky lifestyles.

Since a consistent temporal association between trends in mortality and alcohol consumption was not shown conclusively in all groups (bearing in mind the limitations of the data), it is possible that whilst alcohol may contribute to the excess mortality of the transition, the causes of excess deaths may have been multiple and complex. Other research has also shown rapid changes in economic, employment-related and psychological conditions [22,46]; an independent association of socioeconomic variables with mortality [8,47]; and a rise in deaths from external causes that was only partly attributable to alcohol [13]. Further research is required into the interactions between hazardous drinking and socioeconomic conditions, and the influence of gender. Nevertheless, widespread heavy alcohol consumption remains a major cause of premature mortality and a serious public health issue in Russia [8,9].

## Conclusions

Sudden economic decline in late 1990s Russia was associated with a sharp, temporary fall in heavy drinking, especially amongst the newly unemployed and a gradual and persistent increase in home distilled spirit consumption, with the latter more common amongst disadvantaged groups. The correlation between heavy drinking and national mortality in lower educated men is interesting, but the timing of RLMS surveys late in the calendar year, and the absence of any correlation between drinking and the subsequent year's mortality, makes these data hard to interpret.

The high prevalence of harmful drinking shown here highlights an urgent need for more effective alcohol policies in Russia, particularly to control widespread distillation and consumption of samogon, and to address public fears of counterfeit vodka poisoning [20]. Taxation is unlikely to be effective, since cheaper spirit consumption rose when vodka became less affordable, but engaging individuals and communities to make legislation effective has been proposed as another potentially useful policy measure [48]. The 2006 legislation restricting beer consumption [38] may also have some effect. Ongoing surveys of consumption will need to include a diverse range of alcohols.

### Limitations

The first limitation in this study is the incomplete knowledge of the alcohol content of the beverages studied. Although the content of vodka and samogon was based on chemical analyses [19], the content cannot be entirely certain.

Second, episodic heavy drinking may have been underestimated, since quantity was measured by individual beverage, and could not capture combinations of drinks per occasion [23]. Furthermore, alcohol consumption may be under-reported in Russian surveys [49], and socially marginalised, heavy drinking individuals[50] were clearly not included.

Third, any unequally distributed losses to follow-up (including deaths) could affect the results, either because people with particular characteristics left more frequently, or because heavy drinkers did so. However, there are two reasons why this is unlikely to be the case. First, the panel age composition showed minor changes, confined to the youngest age groups, with a decrease of 4% in males and females aged 30-39, and a corresponding increase in 18-29 year olds, suggesting that any effect on the results would be small. Second, drinking pattern had little effect on leaving the study. In a subset of 6,000 men present in 1996, time in study did not differ significantly by drinking pattern (moderate drinkers 6.6 years; frequent, heavy drinkers 6.8 years). Furthermore, age adjusted logistic regression analyses indicated few differences in male drinkers leaving over the short term. Frequent heavy consumers in 1996 were no more likely to die or leave RLMS without explanation by 1998 than moderate drinkers, and whilst they were more likely to leave through household break-up [1.58 (1.08-2.25)], perhaps reflecting domestic strain [51], the absolute difference appeared insufficient to explain the short-term fall in the prevalence of frequent heavy drinking in 1998.

Despite some limitations, there are several reasons why the data can be reasonably used to assess trends. First, the determinants of reporting would not be expected to vary between years. Second, although it has been claimed that alcohol consumption in RLMS may underestimate total population drinking [52], perhaps for the reasons above [50], consumption in RLMS was consistent with, and often higher than, other Russian surveys, and it could be reasonably concluded that enough heavy drinkers participated in RLMS to assess trends. Whilst some very heavy drinkers could have been missed [53], this is inevitable in any survey, and it would be surprising if major population consumption changes were not visible in a representative population survey sample.

It should also be noted that whilst alcohol measures from this dataset have been studied previously [54,55], the analyses in this paper have a new and substantially different focus, addressing harmful drinking patterns and their determinants, and their link with mortality trends and with the economic changes around the 1998 crash.

## Competing interests

The author declares that they have no competing interests.

## Author information

At the time this research took place, FP was a Clinical Lecturer at the London School of Hygiene and Tropical Medicine.

## Pre-publication history

The pre-publication history for this paper can be accessed here:

http://www.biomedcentral.com/1471-2458/10/691/prepub
